# Comprehensive Study on the Feasibility of Pyrolysis Biomass Char Applied to Blast Furnace Injection and Tuyere Simulation Combustion

**DOI:** 10.1021/acsomega.1c01677

**Published:** 2021-07-27

**Authors:** Han Dang, Guangwei Wang, Chen Wang, Xiaojun Ning, Jianliang Zhang, Xiaoming Mao, Nan Zhang, Chuan Wang

**Affiliations:** † State Key Laboratory of Advanced Metallurgy, University of Science and Technology Beijing, 30 Xueyuan Road, Haidian District, Beijing 100083, China; ‡ Baosteel Research Center, No. 889 Fujin Road, Baoshan District, Shanghai 201900, China; § Swerim AB, Luleå SE-971 25, Sweden; ∥ Thermal and Flow Engineering Laboratory, Åbo Akademi University, Åbo FI-20500, Finland

## Abstract

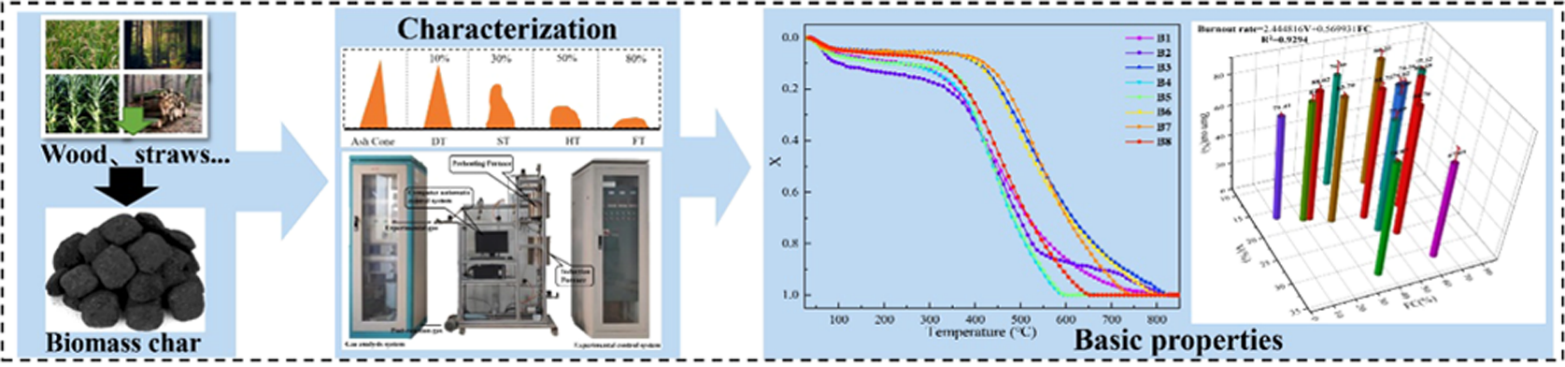

Basic property analysis is the most comprehensive evaluation of metallurgical characteristics of blast furnace injection fuel. In this study, the basic properties of 16 types of pyrolysis biomass char samples were comprehensively investigated; the results showed that components harmful to a blast furnace, such as the ash content and alkali metal content of Jiangsu Suzhou woodblock char (B3), Jiangsu Changzhou branch char (B8), Jiangsu Zhangjiagang bamboo char (B10), and Jiangsu Zhangjiagang coconut shell char (B12) in all of the biomass char samples, are lower and close to the level of blast furnace injection bituminous coal. The grindability, particle size distribution, and safety all met the requirements of the blast furnace. Among them, the ash melting characteristic temperature of B3, B8, Jiangsu Zhangjiagang rice husk char (B11), and Shanghai soil remediation agent (B16) was greater than 1250 °C, indicating that they are not easy to block the blast furnace raceway and spray guns. Most of the biomass char samples had good combustibility, and the burnout temperature was less than 700 °C. A self-developed blast furnace injection combustion simulation experimental device was used to simulate the combustion behavior of biomass char in the blast furnace raceway tuyere, and the burnout rates of 16 biomass chars were measured. The results showed that that the burnout rate is related to both the volatiles and fixed carbon and the influence of volatiles on the burnout rate is greater than that of fixed carbon. The burnout rates of B3 and B8 were 77.12 and 67.03%, respectively. Above all, B3 and B8 showed good properties, but the burnout rate of B3 was higher, so B3 had the feasibility of applying to blast furnace injection, which indicates that woodblock char has the potential to be used as blast furnace injection fuel.

## 1 Introduction

With the rapid increase of carbon dioxide emissions and greenhouse gases, climate change is a global problem faced by mankind and poses a threat to human society and life systems. In this context, countries around the world have put forward the goal of “Carbon Neutrality” by reducing greenhouse gases in a global agreement.^1,2^ In September 2020, China announced that it would increase its nationally determined contributions, adopt more effective policies and measures, and strive to achieve a peak of carbon dioxide emissions by 2030 and achieve Carbon Neutrality by 2060. This is the first time that China has explicitly set a Carbon Neutrality target, and it is also a long-term policy signal of China’s economic transition to low carbon, which has attracted extensive attention from the international community. To achieve the goal of Carbon Neutrality ahead of schedule, BaoSteel, the largest steel producer in the world, has proposed many solutions. One solution is to optimize the energy structure; increase investment in energy-saving and environmental protection technologies; continuously increase the proportion of clean energy such as natural gas; increase the utilization of renewable energy such as solar, wind, and biomass energy; deploy the hydrogen energy industry; and promote a clean and low-carbon energy structure.^3−5^ China is a largely agricultural country with abundant biomass resources; the total amount of agricultural and forestry waste generated every year reaches 1.6 billion tons; if all of this was used as biomass fuel, it can be converted into 800 million tons of standard coal.^6^ Compared with traditional fossil fuels, biomass (waste wood, straw, etc.) is a kind of renewable carbon-neutral energy with wide distribution and high yield, and less sulfide and nitrous compounds were released during burning, which has great environmental protection advantages and social benefits.^7^ If abundant biomass resources were applied to blast furnace ironmaking process production, on the one hand, it can effectively reduce the traditional blast furnace ironmaking technology’s dependence on fossil energy and reduce CO_2_ emissions in steel production, which will help achieve the strategic goal of being carbon-neutral; on the other hand, it can also utilize a large amount of agricultural and forestry wastes, thus reducing the environmental impact of open burning and improving waste utilization efficiency.^8^

Biomass has great potential to be used in blast furnace injection. However, due to large amounts of lignin and cellulose in biomass, it is difficult to crush and has poor grindability. In addition, biomass also has the characteristics of small volume density, low energy density, low combustion efficiency, and high moisture content and volatile (V) content, which make the storage and transportation cost of biomass higher, industrial processing difficult, and limit the application of biomass in blast furnace injection.^9^ Therefore, pretreatment and upgrading treatment are necessary to improve the quality and efficiency of biomass in combustion and gasification applications. Thermochemical pretreatment of biomass is carried out to obtain products that meet the blast furnace fuel standards. Pyrolysis is a biomass pretreatment technology that attracted much attention in recent years, whose process parameters can be targeted optimization. By changing the pyrolysis conditions, products with different compositions and properties can be obtained; after pyrolysis pretreatment, the properties of biomass char can be improved, and the calorific value and grindability can meet the requirements of blast furnace injection fuel. Therefore, pyrolysis plays an important role in improving the fuel characteristics of biomass and reducing transportation and storage costs.^10^

In recent years, a lot of research has carried out on the application of pyrolysis biomass char in blast furnace injection.^11^ Yousaf et al.^12^ studied the combustion characteristics of biomass char obtained from the pyrolysis of peanut shell and wheat stalk at 300, 500, and 700 °C and found that when the mass ratio of biomass char was 50%, the blends mixed with pulverized coal had the highest combustion efficiency. Du^13^ studied the feasibility of pretreatment of bamboo, rice husk, bagasse, and other biomass materials for blast furnace injection and compared the performance of the treated biomass and the two types of high volatile and low ash content used for blast furnace injection. The results showed that the combustion performance of the pretreated biomass material is better than that of low-ash coal, When mixed with anthracite for blast furnace injection, has a better performance. Wang et al.^14^ simulated the effect of replacing pulverized coal injection with pyrolysis wood char on a blast furnace, and the results showed that S and P contents in molten iron had a decreasing trend. If pulverized coal injection was completely replaced, CO_2_ emission could be reduced by 1140 kt per year and energy saving could be 77 GWh. Mathieson et al.^15^ studied the combustion performance of four kinds of wood char and pulverized coal under the condition of simulated blast furnace tuyeres, and the results showed that the combustion performance of wood char was better than that of pulverized coal with high volatile content, and the improvement effect of oxygen enrichment on the combustion performance of wood char was significant. According to the above research results, some current studies mainly focus on the combustion characteristics of biomass, the preparation of biomass char, the assessment of energy consumption and environment, etc., and there is a lack of systematic research on the basic properties (grindability, harmful elements analysis, safety properties, etc.) of biomass char used in blast furnace injection. In addition, there are fewer biomass types in each study, so it is difficult to make a detailed comparison of biomass commonly used in life. Therefore, it is necessary to carry out a comprehensive and systematic study on the basic properties of many kinds of biomass samples to evaluate the metallurgical properties of different kinds of biomass and find out the potential biomass as blast furnace fuel.

A systematic study of the various technical characteristics of 16 types of biomass char samples was carried out in this work to provide guidance for the feasibility of applying biomass char to blast furnace injection. First, the proximate analysis, ultimate analysis, and ash composition and alkali metal composition analysis of biomass char were carried out, and then, the physical and process characteristics of different biomass char samples were analyzed, including grindability, particle size distribution, ignition temperature, explosiveness, ash melting characteristics, calorific value, and combustibility. In addition, the self-developed blast furnace injection combustion simulation experimental device was used to test the burnout rate of different pulverized biomass char samples under oxygen-rich, high-temperature conditions and simulate the combustibility of pulverized biomass char in the blast furnace tuyere raceway. Through the above systematic research, different types of biomass char samples were evaluated, and the biomass char sample that can replace bituminous coal was found, which provides guidance for the green and circular development of blast furnace ironmaking production.

## 2 Material and Methods

### 2.1 Materials

The 16 pyrolysis biomass char samples used in this research were all provided by BaoSteel. They were named as Shenzhen garden biomass char (B1), Jiangsu Zhenjiang biomass char (B2), Jiangsu Suzhou woodblock char (B3), Jiangsu Suzhou garden biomass char (B4), Jiangsu Suzhou cotton straw char (B5), Jilin Songyuan biomass char (B6), Jiangsu Suqian bamboo char (B7), Jiangsu Changzhou branch char (B8), Jiangsu Zhangjiagang cotton straw char (B9), Jiangsu Zhangjiagang bamboo char (B10), Jiangsu Zhangjiagang rice husk char (B11), Jiangsu Zhangjiagang coconut shell char (B12), Jiangsu Zhangjiagang garden biomass char (B13), Jiangsu Zhangjiagang reed bamboo char (B14), Jiangsu Zhangjiagang sawdust char (B15), and Shanghai soil remediation agent (B16).

### 2.2 Basic Properties Analysis

The proximate analysis and ultimate analysis of the samples were carried out according to the GB/T212-2008 standard. The ash content analysis was measured with an X-ray fluorescence (XRF) analyzer. The analysis of alkali metal content was carried out by an ultraviolet–visible spectrophotometer. According to the ultimate analysis results of different samples, the high calorific value (HHV) was calculated by using the following formula, which is defined as^15^
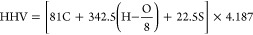
1where C, H, O, and S are the contents of elements C, H, O, and S of different biomass char samples.

The safety properties of the samples include the ignition temperature and explosiveness, which were measured by a solid oxidizer method and a long-tube test device, respectively, and the standard is GB/T18511-2001. In this study, the Hardgrove method was used to determine the grindability index (HGI) of different biomass char samples, which is defined as^16^
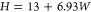
2where *W* is the weight of the pulverized biomass char less than 74 μm after grinding by a Hardgrove grindability tester.

**Figure 1:**
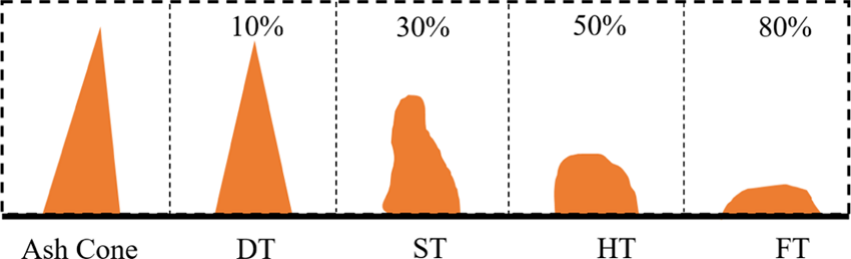
Schematic diagram of different characteristic temperatures of ash fusion.

The particle size distribution of 16 kinds of biomass char samples was measured with an LMS-30 particle size distribution meter. The GB/T219 standard was used to determine the deformation temperature (DT), softening temperature (ST), hemispheric temperature (HT), and flow temperature (FT) of biomass char, as shown in Figure 1. DT, ST, HT, and FT are the temperatures at which the gray cone shrinks 10, 30, 50, and 80%, respectively.^17^ In this study, an HCT-4 microcomputer differential thermal balance produced by Beijing Hengjiu was used to measure the combustibility of different pulverized biomass char samples. The samples were dried for 24 h at 378 K and then ground to less than 74 μm. The fixed carbon (FC) combustion conversion rate (*x*) of the sample was calculated according to the following formula
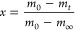
3where *m*_0_ represents the initial mass of the sample, *m*_*t*_ represents the instantaneous mass of the sample at time *t*, and *m*_∞_ is the final mass of the sample, which corresponds to the ash content. The apparent reaction rate was calculated as the ratio of conversion to time expressed as d*x*/d*t*. *S* was defined as the comprehensive combustion characteristic index, which was determined by eq 4
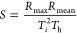
4*R*_max_ represents the maximum conversion rate and *R*_mean_ represents the average conversion rate, s^–1^.

### 2.3 Tuyere Combustion Simulation Experiment

**Figure 2:**
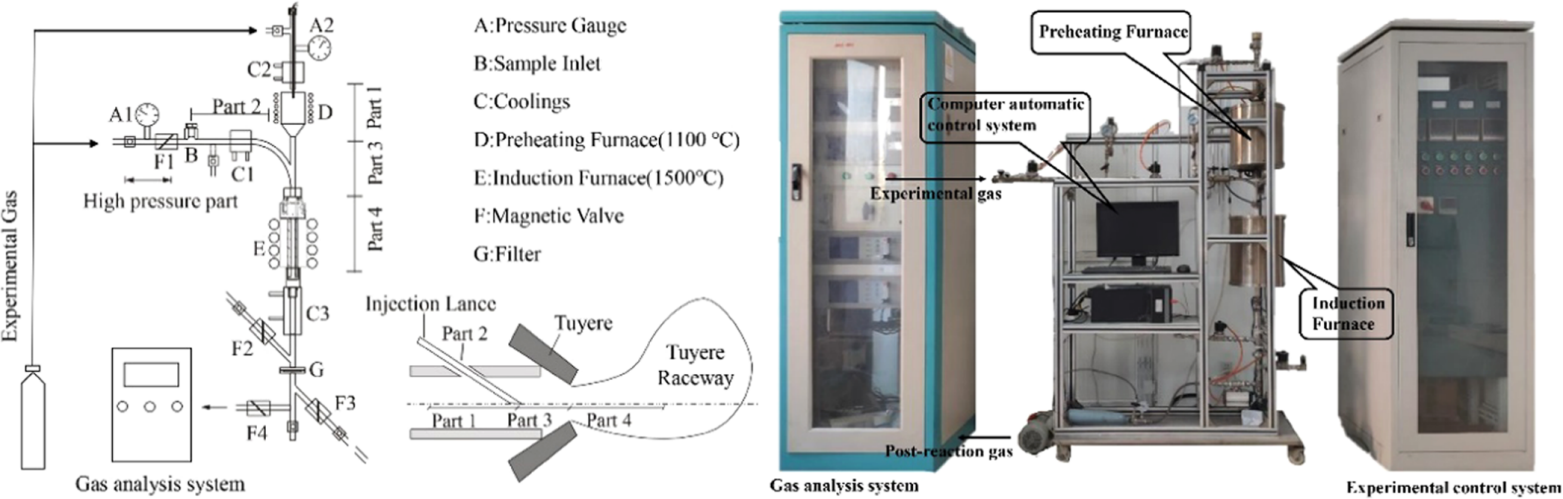
Schematic and physical diagram of the blast furnace coal injection combustion simulation device.

To simulate the combustion behavior of blast furnace injection fuel in the blast furnace tuyere raceway, the self-developed blast furnace injection combustion simulation experimental device was used to test the burnout rates of different biomass char samples under oxygen-rich, high-temperature conditions. The schematic diagram and the physical diagram of the device are shown in Figure 2. The main body of the equipment includes a high-pressure section (simulated oxygen-coal spray gun, 0.4 MPa) and a low-pressure section (simulated hot air and blast furnace tuyere raceway, 0.2 MPa). The linkage device controls the biomass char sample and the simulated hot wind (1100 °C) in the high-temperature furnace (1500 °C), the reacted gas was filtered and then introduced into the gas analyzer, and the burnout rate of biomass char was calculated by inverse calculation of the gas composition. To ensure the accuracy of the experimental results, each group of experiments was carried out five times, the highest value and the lowest value were removed, and the average value was taken as the biomass char burnout rate; a higher burnout rate means more complete combustion in the raceway of the blast furnace during blast furnace injection.

## 3 Results and Discussion

### 3.1 Proximate and Ultimate Analyses of the Biomass Char

**Figure 3:**
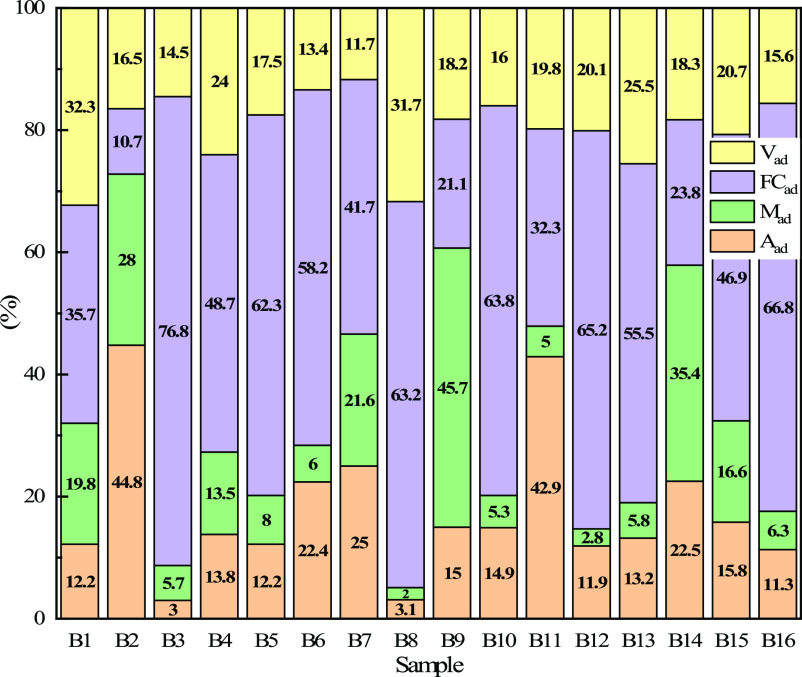
Proximate analysis of different biomass char samples.

Proximate and ultimate analyses are the basic analysis of fuel quality, through which one can understand the chemical composition of the fuel, ash, volatile matter, S content, and high heating value (HHV), which are the key judgment indexes. The results of proximate and ultimate analyses of 16 types of biomass char are shown in Table 1 and Figure 3. It can be seen from Table 1 that the volatile content of all biomass char is similar to that of bituminous coal (10–37%); most of the biomass char samples have higher fixed carbon, lower ash content, lower O and S content. Low ash content is conducive to reducing the coke ratio, and higher fixed carbon will generate more heat, which is conducive to the stable production of the blast furnace.^18^

**Table 1:** Proximate and Ultimate Analyses of Biomass Char

	proximate analysis (%)				ultimate analysis (%)					
sample	M_ad_	A_ad_	V_ad_	FC_ad_^a^	C	H	O^a^	N	S	HHV (MJ/kg)
B1	19.8	12.2	32.3	35.7	65.07	3.34	18.23	0.84	0.32	23.62
B2	28.0	44.8	16.5	10.7	51.99	1.55	0.56	0.60	0.49	19.80
B3	5.7	3.0	14.5	76.8	87.35	2.84	6.38	0.29	0.14	32.57
B4	13.5	13.8	24.0	48.7	64.88	2.94	16.44	1.59	0.35	23.30
B5	8.0	12.2	17.5	62.3	68.58	2.43	15.21	1.24	0.34	24.04
B6	6.0	22.4	13.4	58.2	63.25	1.99	11.81	0.42	0.13	22.20
B7	21.6	25.0	11.7	41.7	69.03	1.58	3.87	0.41	0.11	24.99
B8	2.0	3.1	31.7	63.2	72.32	3.55	19.63	1.31	0.09	26.12
B9	45.7	15.0	18.2	21.1	61.31	2.30	19.92	1.22	0.25	20.54
B10	5.3	14.9	16.0	63.8	73.00	2.58	8.54	0.82	0.16	26.95
B11	5.0	42.9	19.8	32.3	42.65	2.37	11.42	0.51	0.15	15.82
B12	2.8	11.9	20.1	65.2	49.69	2.12	35.23	0.61	0.45	13.64
B13	5.8	13.2	25.5	55.5	56.22	2.63	26.07	1.39	0.40	18.21
B14	35.4	22.5	18.3	23.8	40.18	1.94	34.74	0.48	0.16	10.20
B15	16.6	15.8	20.7	46.9	68.32	2.48	12.07	1.12	0.21	24.59
B16	6.3	11.3	15.6	66.8	70.62	2.45	14.95	0.51	0.17	24.80

a Calculated by difference. M, moisture; FC, fixed carbon; A, ash; V, volatile matter; ad, air dry basis.

Too much moisture will increase storage, transportation, and grinding costs; it will not only reduce its calorific value but also absorb the heat in the blast furnace.^19^ As shown in Figure 3, the moisture content of B2, B7, B9, and B14 is higher than 20%, and the moisture contents of B3, B5, B6, B8, B10, B11, B12, B13, and B16 are 5.7, 8.0, 6.0, 2.0, 5.3, 5.0, 2.8, 5.8, and 6.3%, respectively. Nearly half of biomass char samples’ ash content was less than or approximately equal to 10%. Among them, the ash contents of B1, B3, B5, B8, B12, B13, and B16 were 12.2, 3.0, 13.8, 12.2, 3.1, 11.9, 13.2, and 11.3%, respectively, and about 10% of the ash content meets the requirements of blast furnace production.^20,21^ Volatile matter represents the metamorphism of biomass char, and the content of volatile matter will affect the safety during transportation and storage. It can be seen that the volatile content of all biomass char samples is within the range of 10–37%, reaching the level of bituminous coal (10–37%), which indicates biomass char samples have the same potential as bituminous coal for blast furnace injection.^22,23^ The fixed carbon difference between different samples can reach about 66%. Among them, the fixed carbon content of B3, B5, B6, B8, B10, B12, B13, and B16 is above 50%, and its values are 76.8, 62.3, 58.2, 63.2, 63.8, 65.2, 55.5, and 66.8%, respectively, which can provide more heat when used for blast furnace injection.

**Figure 4:**
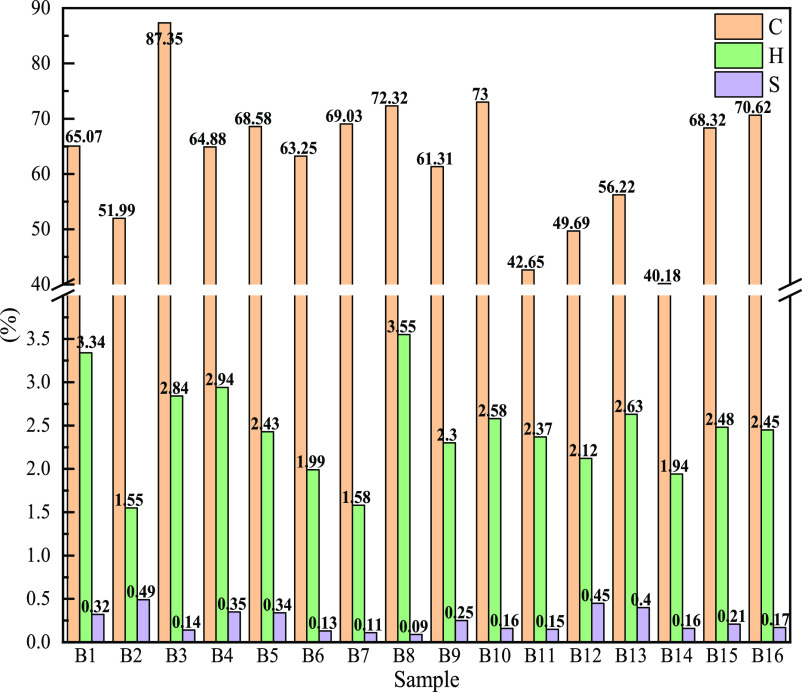
Ultimate analysis of different biomass char samples: C, H, and S.

As shown in Figure 4, most of the C content of biomass char is basically above 60%, which meets the requirements of blast furnaces. The hydrogen content is between 1 and 4%, slightly lower than that of common bituminous coal. C and H are the main combustible elements in biomass char, which emit a lot of heat when burnt.^24,25^ Sulfur is an element that is extremely harmful to blast furnace ironmaking. An increase in sulfur content requires more solvents for desulfurization, which will consume more heat and coke, thereby affecting the cost of iron per ton.^26^ The S content of all biomass char samples is less than 0.5%, in line with the quality requirements for blast furnace injection,^27^ which have little impact on the blast furnace.

**Figure 5:**
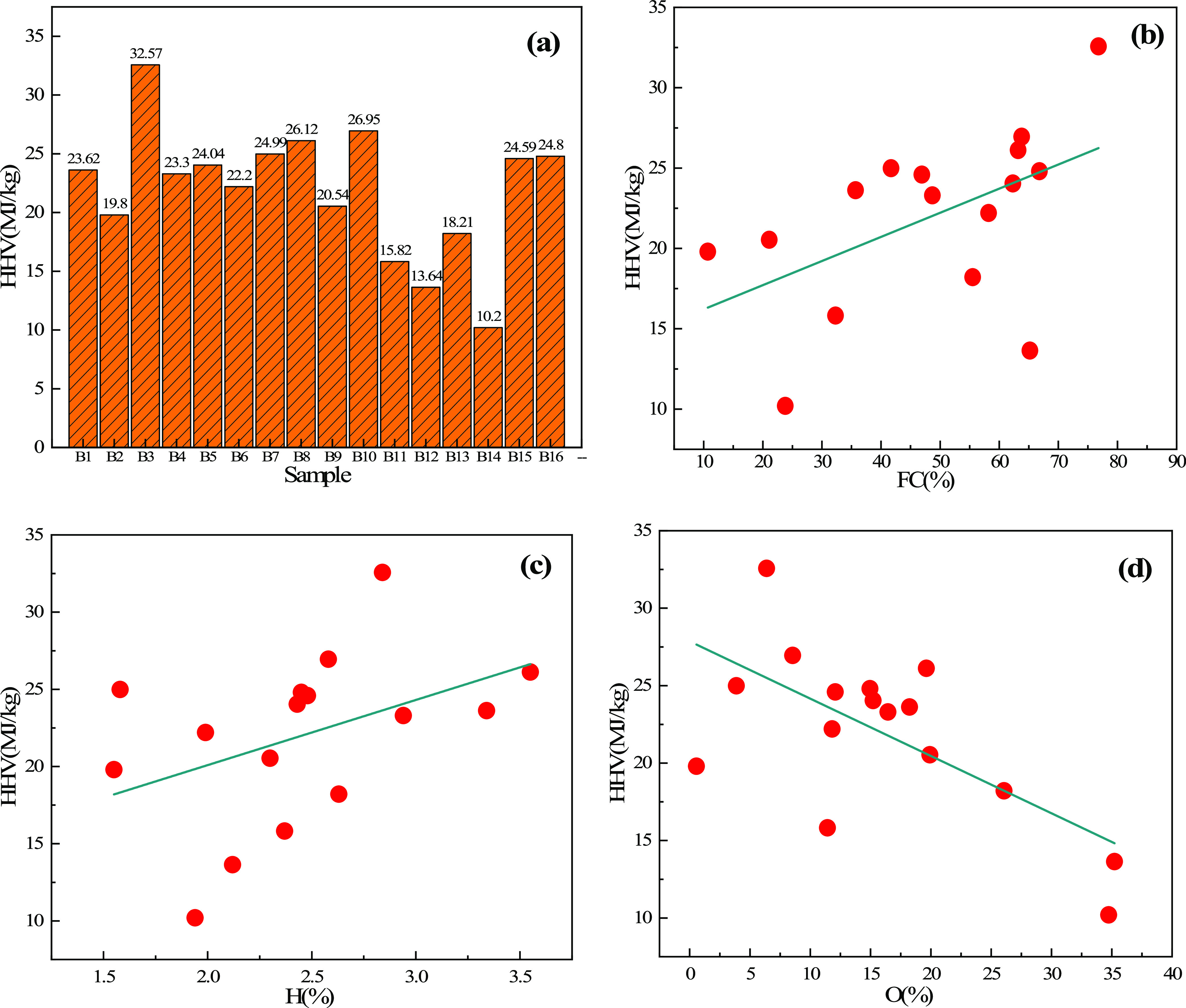
(a) HHVs of different biomass char samples, (b) relationship between FC and HHV, (c) relationship between H and HHV, and (d) relationship between O and HHV.

The high heating value (HHV) of coal refers to the heat emitted by the combustion of a unit of coal.^28^Figure 5a shows the HHVs of 16 types of biomass char samples. It can be seen that the HHVs of different biomass char samples are quite different. The HHV of most of the biomass char samples is above 20 MJ/kg, which is close to that of thermal coal such as lignite. The largest is B3 (wood char) with a HHV of 32.57 MJ/kg, followed by B10 (bamboo char) with a calorific value of 26.95 MJ/kg, reaching the standard of anthracite (27–37 MJ/kg). The relationship between FC, H, and O content and the calorific value of different biomass char samples is shown in Figure 5b–d. It can be seen that the HHV of biomass char shows a trend of increasing with an increase of FC content and H content; B3 with the highest FC content has the highest calorific value. With an increase of O content, the HHV shows a downward trend.

In general, B3, B5, B8, and B16 have relatively high HHV, low ash content, and moderate volatile matter, which meets the requirement of blast furnace fuel injection.

### 3.2 Ash Composition and Ash Melting Characteristics of Biomass Char

Ash composition and ash melting characteristics are important factors to judge the quality of blast furnace injection fuel; inorganic substances in ash can affect the fluidity of blast furnace slag, desulfurization capacity, and ash melting point of the fuel.^29,30^ A too low ash melting point will accelerate the accumulation and deposition of biomass char, easy to lead to the tuyere or before the spray gun slagging, and will also prevent oxygen from entering the unburned biomass char, reducing combustion efficiency. A too high ash melting point will affect the furnace desulfurization and slag discharge.^30^

**Figure 6:**
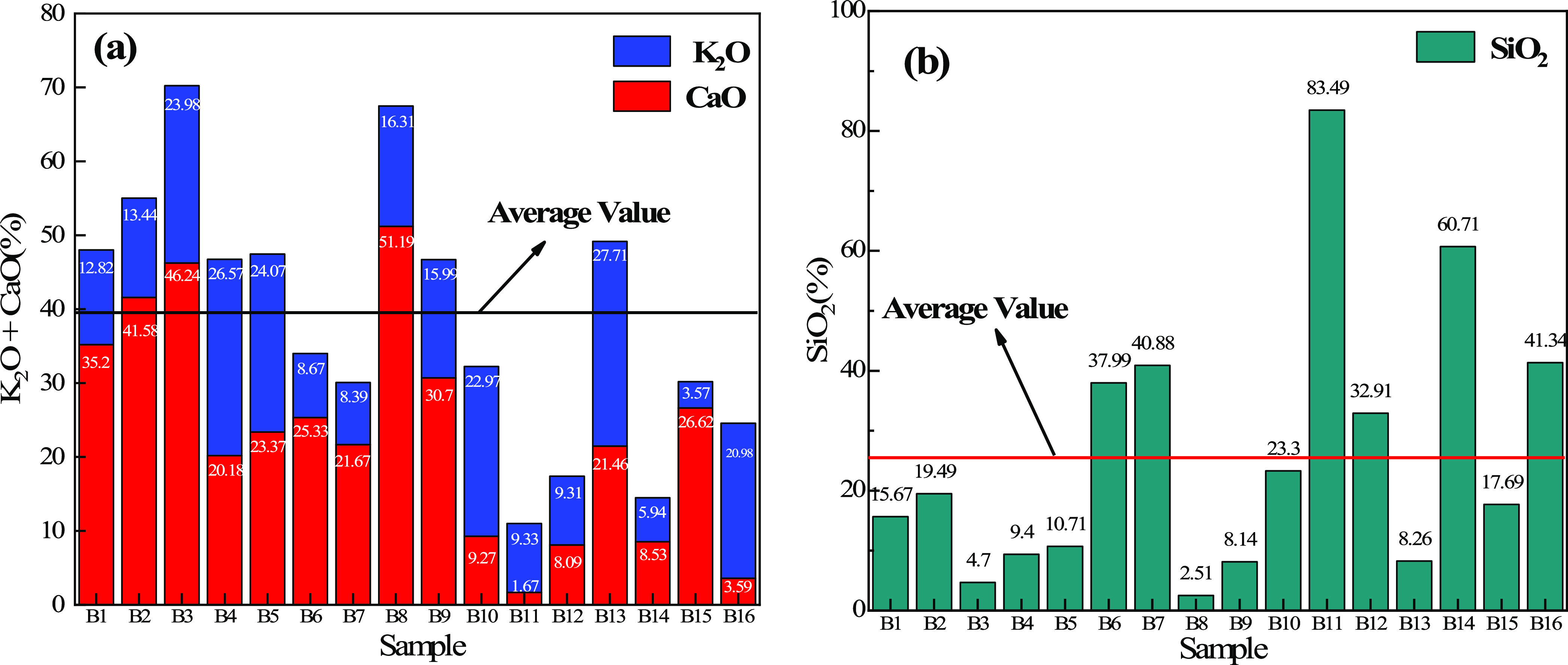
(a) Content distribution of CaO and K_2_O in ash and (b) content distribution of SiO_2_ in ash.

The ash contents of different biomass char samples are shown in Table 2. It can be seen that the ash contents of biomass char samples were mainly composed of SiO_2_, Al_2_O_3_, CaO, MgO, and K_2_O. The contents of CaO, MgO, and K_2_O in the ash of most biomass char samples are high, which makes most of them produce alkaline slag when used for blast furnace injection. Figure 6 shows the contents of SiO_2_, CaO, and K_2_O of different biomass char ash samples; due to the high SiO_2_ content in B6, B7, B11, B12, B14, and B16, these biomass char samples will form acidic slag, while the rest will form alkaline slag and an acidic solvent will be needed to be added to reduce the alkalinity.

**Table 2:** Ash Composition of Different Samples (wt %)

sample	SiO_2_	Al_2_O_3_	Fe_2_O_3_	CaO	K_2_O	MgO	P_2_O_5_	SO_3_
B1	15.67	6.37	6.29	35.20	12.82	8.07	3.11	2.66
B2	19.49	4.78	8.37	41.58	13.44	4.03	0.19	6.11
B3	4.70	1.39	2.79	46.24	23.98	10.03	5.32	4.57
B4	9.40	4.11	7.64	20.18	26.57	13.4	7.65	5.56
B5	10.71	3.89	3.93	23.37	24.07	15.73	8.29	4.99
B6	37.99	9.95	4.29	25.33	8.67	6.28	2.09	4.19
B7	40.88	11.03	4.45	21.67	8.39	6.08	1.76	4.61
B8	2.51	0.73	8.17	51.19	16.31	11.21	5.85	3.01
B9	8.14	2.91	6.40	30.70	15.99	18.00	10.64	6.49
B10	23.30	7.70	9.21	9.27	22.97	9.78	3.22	4.10
B11	83.49	0.13	0.38	1.67	9.33	0.82	1.89	2.12
B12	32.91	20.05	9.42	8.09	9.31	7.13	2.41	4.66
B13	8.26	4.04	6.68	21.46	27.71	12.92	7.72	5.55
B14	60.71	9.06	5.95	8.53	5.94	3.36	2.95	2.51
B15	17.69	4.40	11.11	26.62	3.57	26.29	1.85	6.30
B16	41.34	10.69	4.24	3.59	20.98	7.12	3.60	6.19

**Figure 7:**
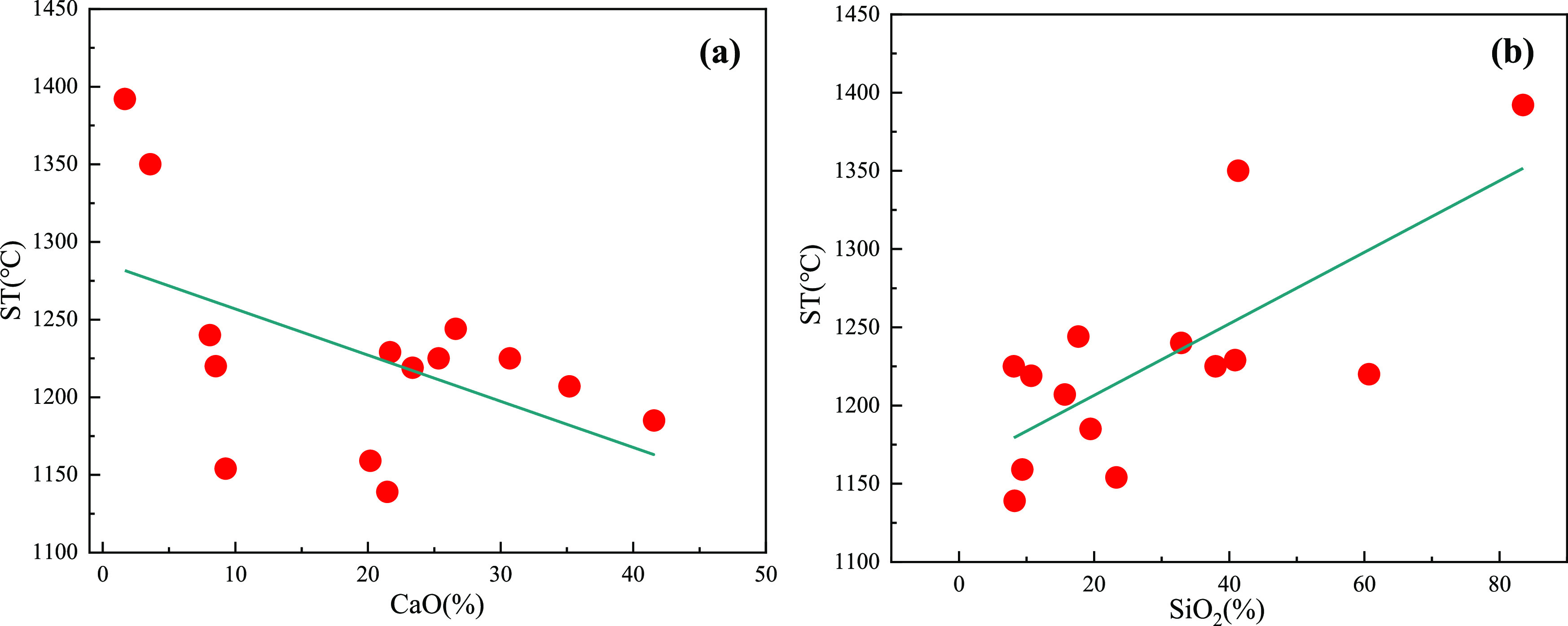
(a) Relationship between softening temperature (ST) and CaO and (b) relationship between softening temperature (ST) and SiO_2_.

Table 3 shows the ash melting characteristics of different biomass char samples; it can be found that the softening temperature of most of the biomass char samples is between 1150 and 1250 °C, which indicates that they have a lower slagging characteristic temperature. When used for blast furnace injection, they may easily build up slag in front of the tuyere or spray gun and block the gun. In addition, the softening temperature of B3, B8, B11, and B16 is greater than 1250 °C, so when used in blast furnace injection, these types of biomass char are not easy to cause slagging on the spray gun. The ash melting point is closely related to the contents of CaO and SiO_2_ in the ash. If the CaO content is high, the ash melting point will be low, and if the SiO_2_ content is high, the ash melting point will be high. The relationship between softening temperature and CaO and SiO_2_ is shown in Figure 7. It can be seen that as the content of CaO increases, the overall softening temperature shows a continuous downward trend, and as the content of SiO_2_ increases, the overall softening temperature shows an increasing trend. Because the ash melting characteristic temperature of most of the biomass char samples is lower than the blast furnace hot air temperature (1250 °C), it is necessary to choose the biomass char with ash melting characteristic temperature higher than 1250 °C to avoid the phenomenon of slag hanging from coal gun.^38^

**Table 3:** Ash Melting Characteristics of Different Biomass Char Samples

sample	deformation temperature (DT)	softening temperature (ST)	hemispheric temperature (HT)	flow temperature (FT)
B1	1180	1207	1212	1224
B2	1162	1185	1196	1211
B3	>1400	>1400	>1400	>1400
B4	1131	1159	1171	1190
B5	1194	1219	1226	1243
B6	1196	1225	1239	1258
B7	1197	1229	1236	1254
B8	1333	>1400	>1400	>1400
B9	1164	1225	1230	1239
B10	1079	1154	1181	1229
B11	1243	1392	>1400	>1400
B12	1200	1240	1252	1291
B13	1125	1139	1150	1165
B14	1192	1220	1236	1259
B15	1231	1244	1247	1258
B16	1285	1350	1370	>1400

### 3.3 Alkali Metal Content of Biomass Char

**Figure 8:**
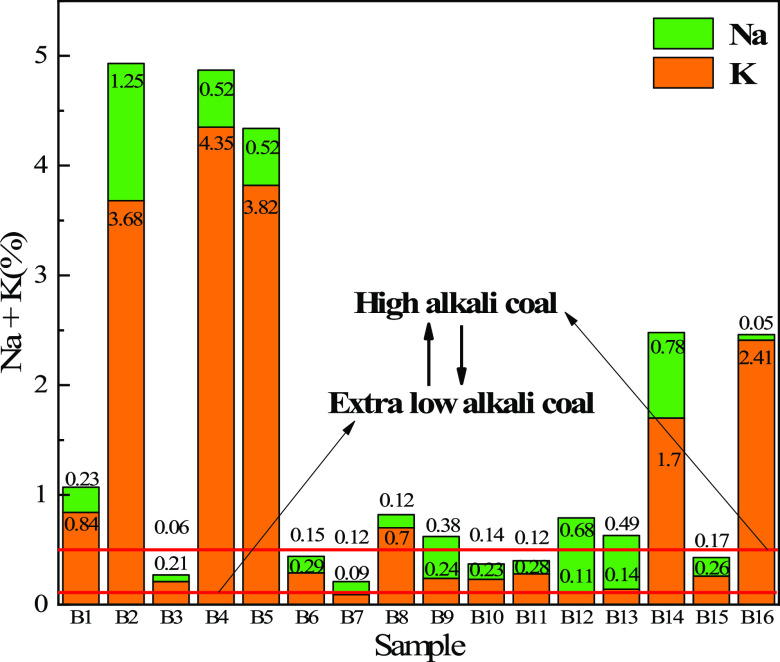
Alkali metal distribution of different biomass char samples.

Table 4 and Figure 8 show the contents of Na and K in different biomass char samples. For blast furnace production, the presence of alkali metals will destroy the quality of coke, and cause the hearth and bottom of the furnace to increase the corrosion rate, and cause the blast furnace nodulation.^31^ It can be seen that most of the biomass char samples have a higher content of K, and only a few biomass char samples have a higher Na content; the total alkali metal content of biomass char basically exceeded 0.1%, and for some samples, it exceeded 0.5%. Therefore, the biomass char samples used in this study all belonged to the level of low-alkali coal (0.1–0.3%) to medium-alkali coal (0.3–0.5%). Among them, the alkali content of B1, B2, B4, B5, B9, B12, B13, B14, and B16 is equal to the content of high-alkali coal, and the alkali metal of these biomass char samples is mainly K element. The biomass char samples with low alkali metal content are B3, B6, B7, B8, B10, B11 and B15, and their alkali metal contents are 0.27, 0.44, 0.21, 0.19, 0.37, 0.40, and 0.43% respectively, which meet the requirements of the blast furnace for low alkali metal content of injection fuel.

**Table 4:** Na and K Contents of Different Biomass Char Samples (wt %)

sample	B1	B2	B3	B4	B5	B6	B7	B8
K (%)	0.84	3.68	0.21	4.35	3.82	0.29	0.09	0.07
Na (%)	0.23	1.25	0.06	0.52	0.52	0.15	0.12	0.12

sample	B9	B10	B11	B12	B13	B14	B15	B16
K (%)	0.24	0.23	0.28	0.11	0.14	1.70	0.26	2.41
Na (%)	0.38	0.14	0.12	0.68	0.49	0.78	0.17	0.05

### 3.4 Safety Properties of Biomass Char

For fuel injection in the blast furnace, pulverized coal with too low ignition temperature is easy to spontaneous combustion, which is one of the main causes of safety accidents such as pulverized coal explosion in the process of preparation, transportation, and injection.^32^ Therefore, to explore the safety of biomass char, the ignition temperature of biomass char was measured. In addition, the explosiveness of biomass char is also closely related to on-site safety production; it has to be considered systematically from pipeline process design to on-site operation.^33,34^

**Figure 9:**
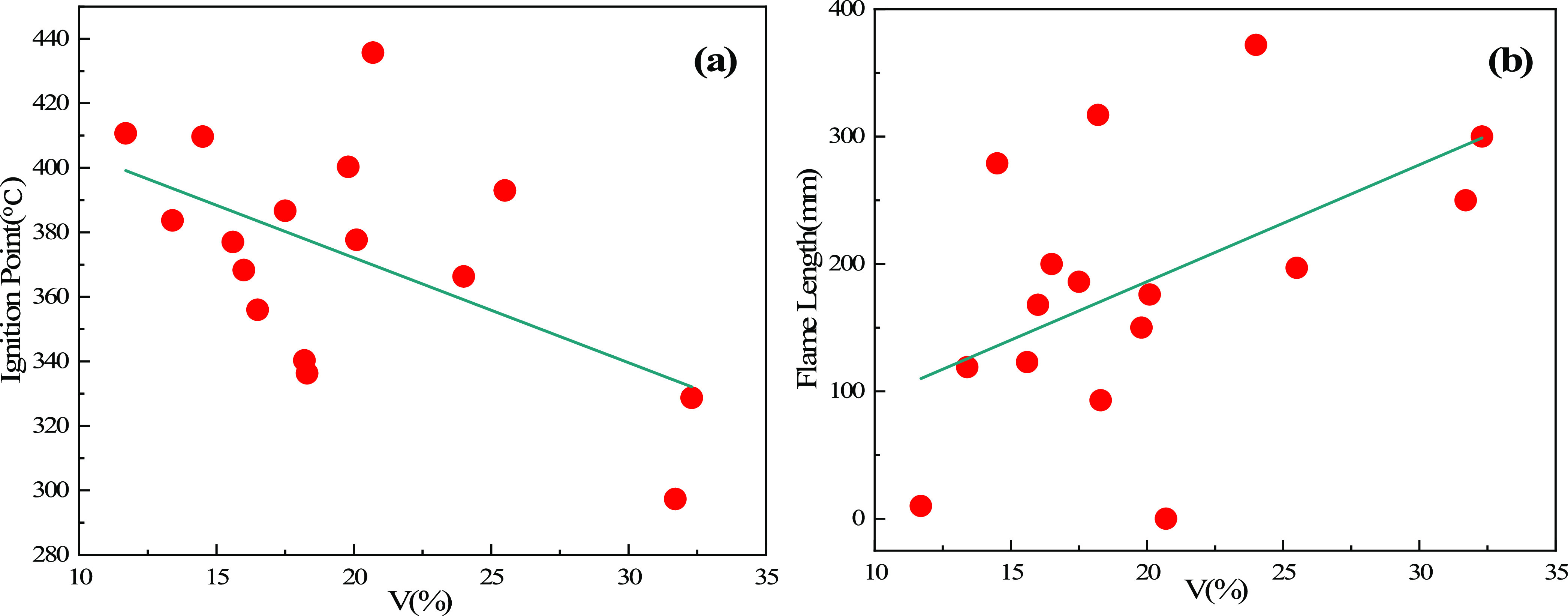
(a) Relationship between ignition temperature and volatile matter and (b) relationship between the explosiveness of biomass char and volatile matter.

The ignition temperature and explosive test results are shown in Table 5. The ignition temperature of most biomass char is between 300 and 400 °C, which is safer for milling, conveying, and blowing. In addition, the flame return lengths of all biomass char samples are less than 400 mm, reaching a weak explosive level. The flame return lengths of the three types of biomass char samples B1, B4, and B9 are between 100 and 300 mm, and the flame return lengths of B3, B6, and B11 are less than 100 mm, which are safer when used for injection. Figure 9a shows the relationship between ignition temperature and volatile matter. With an increase of volatile matter, the ignition temperature gradually decreases. Therefore, biomass char with high volatile matter is more likely to burn when used for injection. Figure 9b shows the relationship between the explosiveness of biomass char and volatile matter. It can be seen that as the volatile matter increases, the explosiveness of pulverized biomass char gradually increases. Therefore, when choosing blast furnace injection fuel, it is necessary to comprehensively consider the volatile matter, ignition temperature, and explosiveness.

**Table 5:** Ignition Temperature and Explosiveness of Different Biomass Char Samples

sample	B1	B2	B3	B4	B5	B6	B7	B8
ignition point (°C)	328.7	356.0	409.7	366.3	386.7	383.7	410.7	297.3
flame return length (mm)	300	150	93	372	168	0	123	200

sample	B9	B10	B11	B12	B13	B14	B15	B16
ignition point (°C)	340.3	368.3	400.3	377.7	393.0	336.3	435.7	377.0
flame return length (mm)	317	176	10	119	279	250	186	197

### 3.5 Grindability and Particle Size Distribution of Biomass Char

Blast furnace injection fuel is usually ground and pulverized before it is used in blast furnace injection to facilitate the combustion of blast furnace injection fuel in the raceway of the tuyere. The grindability index of biomass char indicates the difficulty of crushing biomass char; the lower the grindability index, the greater the energy consumption when used for grinding.^35,36^ The particle size of biomass char has a direct influence on its microstructure, which is an important factor to determine the mass-transfer and heat-transfer characteristics of biomass char. Particle refinement of biomass char can increase the specific surface area, improve its surface activity, and accelerate the combustion rate. Therefore, the particle size of blast furnace for injection fuel is generally required to be less than 0.074 mm and the particle size ratio reaches 70–80%.^37^

**Figure 10:**
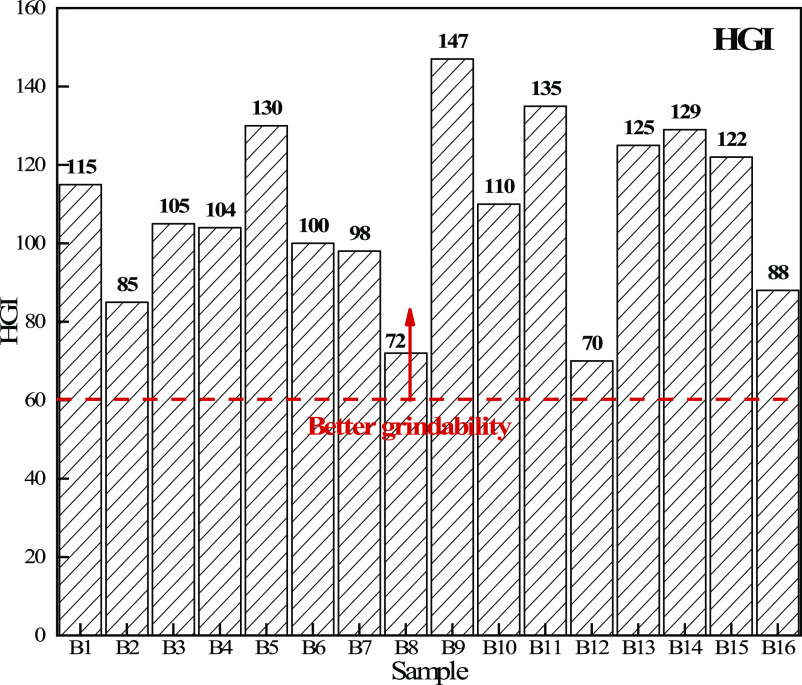
Grindability index distribution of different biomass char samples.

**Figure 11:**
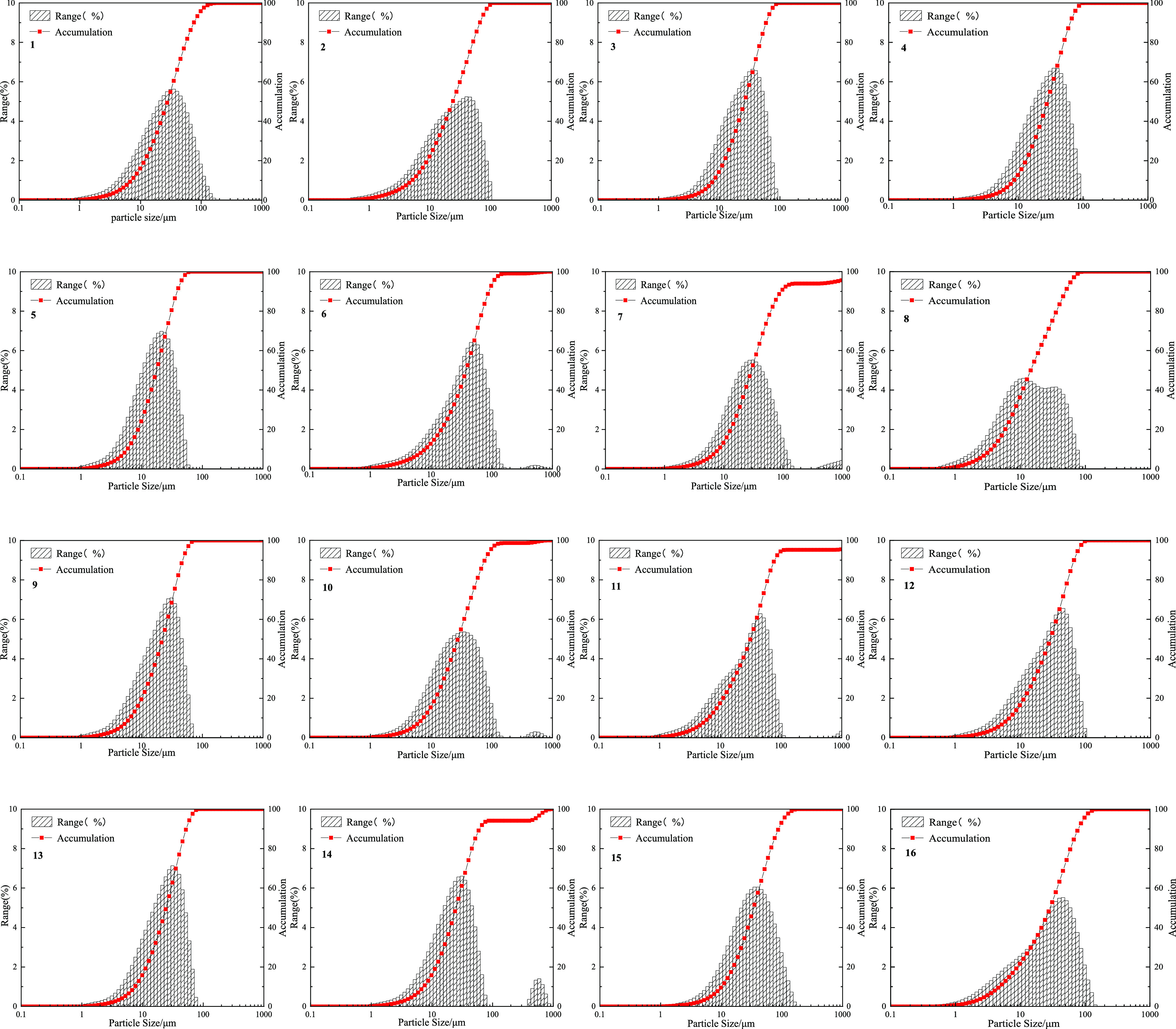
Particle size distribution of different biomass char Samples.

Table 6 shows the grindability index of different biomass char samples. For coal, the grindability index of coal for blast furnace injection should be higher than 60. It can be seen that the grindability index of all biomass char samples is above 60, which meets the conditions for blast furnace fuel injection. Figure 10 shows the grindability index distribution of different biomass char samples. It can be seen that the grindability of B5, B9, and B11 is the best, while the grindability of B2, B8, and B12 is relatively poor, but in general, they meet the grindability requirement of blast furnace injection fuel. Figure 11 shows the particle size distribution of different biomass char samples. From the particle size analysis results, it can be concluded that the particle size of these 16 types of biomass char samples is small, and the proportion of the particle size less than 74 μm is more than 80%, meeting the requirements of blast furnace injection for fuel particle size.

**Table 6:** Grindability Index of Different Biomass Char Samples

sample	B1	B2	B3	B4	B5	B6	B7	B8	B9	B10	B11	B12	B13	B14	B15	B16
HGI	115	85	105	104	130	100	98	72	147	110	135	70	125	129	122	88

### 3.6 Thermogravimetric Analysis of Biomass Char

For blast furnace coal injection, the combustion performance of pulverized coal is an important indicator to measure the combustion status of the blast furnace tuyere. Strengthening the combustion of pulverized coal at the tuyere is the most basic prerequisite for large injection in the blast furnace.^39,40^ Similarly, when biomass char was applied to blast furnace injection, its combustibility determines whether it can be burned completely in the tuyere area when injected into the blast furnace.^42^ However, whether the biomass char is completely burned is not only related to the type, composition, and particle size of the biomass but also closely related to the O_2_ concentration in the blast and the wind temperature.^41^

**Figure 12:**
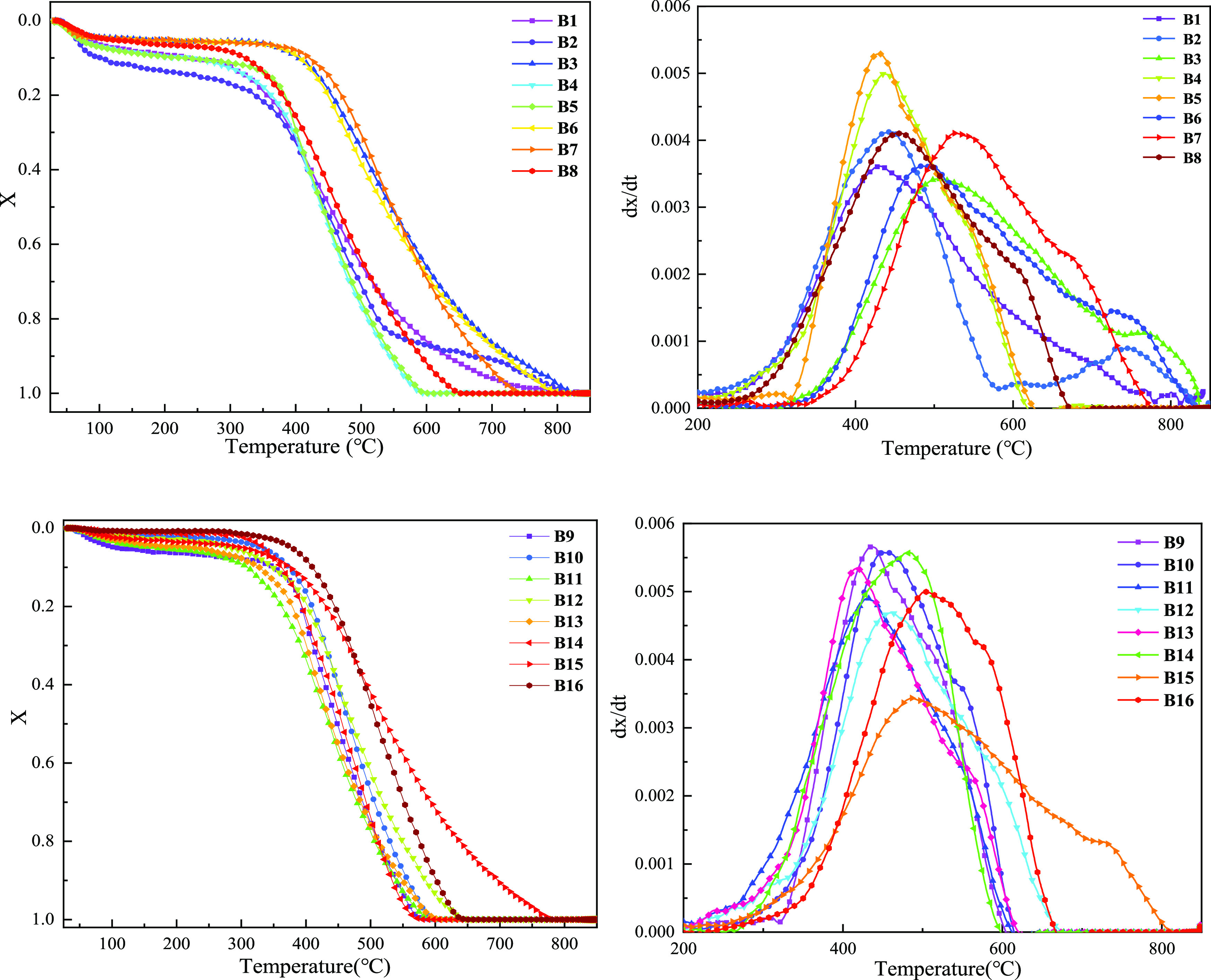
Conversion rate and reaction rate curve of different biomass char samples at the heating rate of 20 °C/min.

The weight loss curve (TG) and weight loss rate curve (DTG) of different biomass char samples are shown in Figure 12. It can be seen that the shapes of reaction curves of different biomass char samples are basically the same. According to its TG curve, the combustion process of biomass char can be divided into the following three stages: (1) dehydration and degassing stage about 25–200 °C. Due to the low temperature of biomass char, there was no drastic chemical reaction in the sample when the temperature was increased. When the temperature of the sample reached about 50 °C, water began to precipitate and the conversion curve began to decline. With an increase of its own temperature, the weight of biomass char slightly increased, the main reason might be the physical and chemical adsorption of the reaction gas on the outer surface and the inner surface of the pore. (2) Sample combustion stage at about 200–600 °C. Biomass char began to lose weight rapidly, volatiles and fixed carbon started to fire and burn successively and soon reached a higher combustion rate, and the maximum weight loss peak appeared in the curve of weight loss rate. With the depletion of combustible materials in the biomass char, the combustion weight loss rate decreased rapidly, and the conversion rate curve gradually flattened. (3) Burnout stage of biomass char from 600 to 800 °C. It can be seen that most of the biomass char had burned out before 700 °C, the conversion curve of biomass char powder remained basically at the level in the burnout stage, and the remaining substances were mainly ash.

For B1–B8, their reaction curves basically show the same trend, and the local maximum weight loss rate occurs at about 100 °C. This stage may be due to the constant precipitation of water and because its own volatiles and fixed carbon have not started to burn. B2 displayed two platforms due to the two peaks in the DTG curve; this may be because B2 has high volatile content and precipitation of water after volatile combustion began; however, at the same time, its ash content is higher, and the residual ash from biomass char burning may precipitate on the pore structure and block its fixed carbon exposed to air, so the combustion reaction occurs at about 500–700 °C; B2 presents the form of slow combustion. When the temperature is greater than 700 °C, the reaction rate of B2 is further increased. This may be due to the decomposition of a certain component in the ash, which makes the residual fixed carbon contact with air, which further enhances the combustion of B2. For B9–B16, their moisture content is not high, so they lose less weight at the initial stage of heating. Most of the combustion range is between 300 and 700 °C, which is in line with the blast furnace’s injection fuel combustibility requirements.

**Figure 13:**
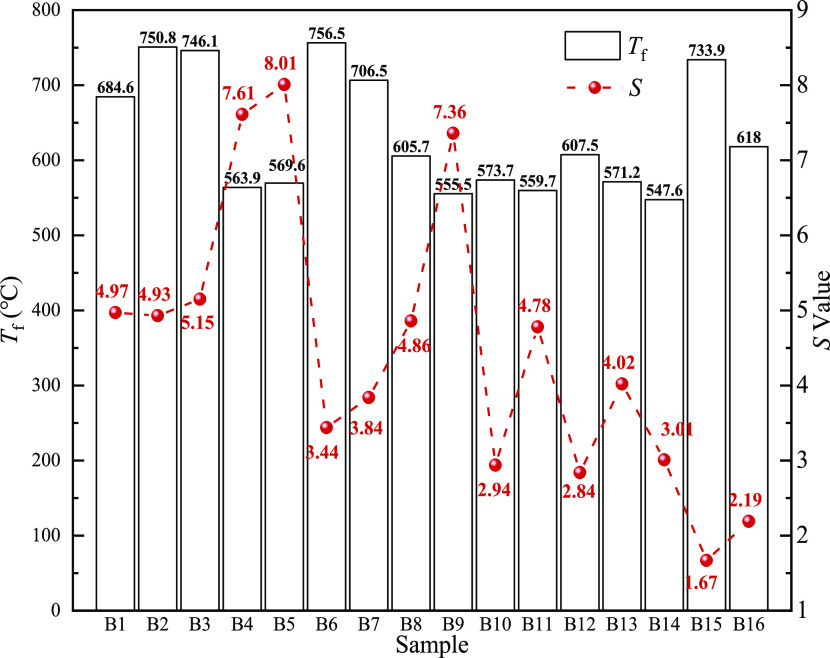
*T*_f_ and *S* of different biomass char samples.

To further study the combustion characteristics of different biomass char samples, the combustion characteristics parameters of all biomass char samples are listed in Table 7. The temperature corresponding to the combustion rate of biomass char reaches 5% in the combustion process and is called the initial combustion temperature (*T*_i_), the temperature corresponding to the combustion rate reaches 95% and is called the burnout temperature (*T*_f_), and the temperature corresponding to the maximum reaction rate in the combustion process is *T*_m_. The maximum reaction rate (*R*_max_) and the average reaction rate (*R*_mean_) are obtained by integrating the reaction curve. The comprehensive combustion characteristic index *S* fully reflects the ignition and burnout characteristics of injection fuel. The larger the *S* value, the better the combustion characteristics of the injection fuel. The time taken for the initial combustion temperature to increase to the burnout temperature is defined as *t*_g_; the smaller the *t*_g_, the shorter the time it takes for the biomass char to burnout.^43,44^ It can be seen from Table 7 that the *S* values of B3, B4, B5, and B9 are greater than 5, which are 5.15, 7.61, 8.01, and 7.36, respectively, indicating that they have good combustion characteristics. The *S* values of the other biomass char samples are less than 5, which shows that there is no definite relationship between the *S* value and *t*_g_ value. For example, B5 has the largest *S*, but its *t*_g_ is not the smallest, and B15 has the smallest *S*, but its *t*_g_ is not the largest. The initial combustion temperature of most biomass char is lower than 200 °C, and the corresponding *t*_g_ is also larger. Among them, B3 has the lowest initial combustion temperature and the largest *t*_g_, which are 58.9 °C and 34.36 min, respectively. According to Table 1, it can be seen that B3 has the highest fixed carbon and low volatile contents. Therefore, a possible reason for its high *t*_g_ value is that there is more fixed carbon and it takes more time to burn up. The initial combustion temperature of B16 is the highest and its *t*_g_ is also the smallest, 378.3 °C and 11.99 min, respectively. The initial combustion temperature and *t*_g_ of B14 were 336.1 °C and 10.56 min, respectively, indicating that these two kinds of biomass char samples begin to react violently after reaching the initial combustion temperature. Table 1 shows that B14 and B16 has lower fixed carbon and volatile; it further explains that the biomass char carbonization degree is one of the factors that may affect the combustion efficiency; the higher the carbonization, the poor the combustion performance. Figure 13 shows the burnout temperatures of different biomass char samples; it can be seen that B2, B3, B6, B7, and B15 have burnout temperatures greater than 700 °C, which may also be due to their high ash content, leading to insufficient contact between their fixed carbon and air and low combustion efficiency. Some of them have high ash content and some have high fixed carbon content; the high *T*_f_ value is caused by more burning of fixed carbon or the possible obstruction of the contact between flammable substances and air by the ash content. However, the complete combustion of biomass char in actual production is not only related to the type, composition, and particle size of biomass but also closely related to the O_2_ concentration and hot wind temperature (1250 °C).

**Table 7:** Combustion Characteristic Parameter of Different Biomass Char Samples at 20 °C/min^a^

sample	heating rate (°C/min)	*T*_i_ (°C)	*T*_m_ (°C)	*T*_f_ (°C)	*R*_max_ × 10^4^ (s^–1^)	*R*_mean_ × 10^4^ (s^–1^)	*S* × 10^6^	*t*_g_ (min)
B1	20	82.9	430.9	684.6	35.97	16.68	4.97	30.09
B2	20	65.4	462.7	750.8	40.70	14.19	4.93	34.27
B3	20	58.9	509.4	746.1	34.72	16.65	5.15	34.36
B4	20	70.9	436.2	563.9	49.91	15.10	7.61	24.65
B5	20	70.1	431.4	569.6	52.89	15.02	8.01	24.98
B6	20	126.8	488.9	756.5	36.14	15.67	3.44	31.49
B7	20	142.7	627.0	706.5	41.04	15.82	3.84	28.19
B8	20	114.0	457.3	605.7	41.04	15.60	4.86	24.59
B9	20	106.9	436.0	555.5	56.59	15.55	7.36	22.43
B10	20	331.1	451.4	573.7	55.74	16.30	2.94	12.13
B11	20	167.6	431.3	559.7	49.06	15.76	4.78	19.61
B12	20	278.9	460.8	607.5	46.89	16.20	2.84	16.43
B13	20	228.2	418.1	571.2	53.48	15.91	4.02	17.15
B14	20	336.1	483.9	547.6	55.73	16.47	3.01	10.56
B15	20	301.3	486.6	733.9	34.37	16.17	1.67	21.63
B16	20	378.3	503.5	618.0	49.99	16.54	2.19	11.99

a Note: *T*_i_, initial combustion temperature; *T*_m_, corresponding temperature of the peak reaction rate; *T*_f_, total combustion temperature; *R*_max_, maximum combustion rate; *R*_mean_, mean combustion rate; *S*, comprehensive combustion index; *t*_g_, time zone of *T*_i_–*T*_f_.

In general, B1–B5, B8, B9, B11, and B13 have large *S* values, which indicates that they have good combustion characteristics and excellent performance when applied to blast furnace injection.

## 4 Simulation Combustion Experiment of Biomass Char in the Blast Furnace Tuyere

**Figure 14:**
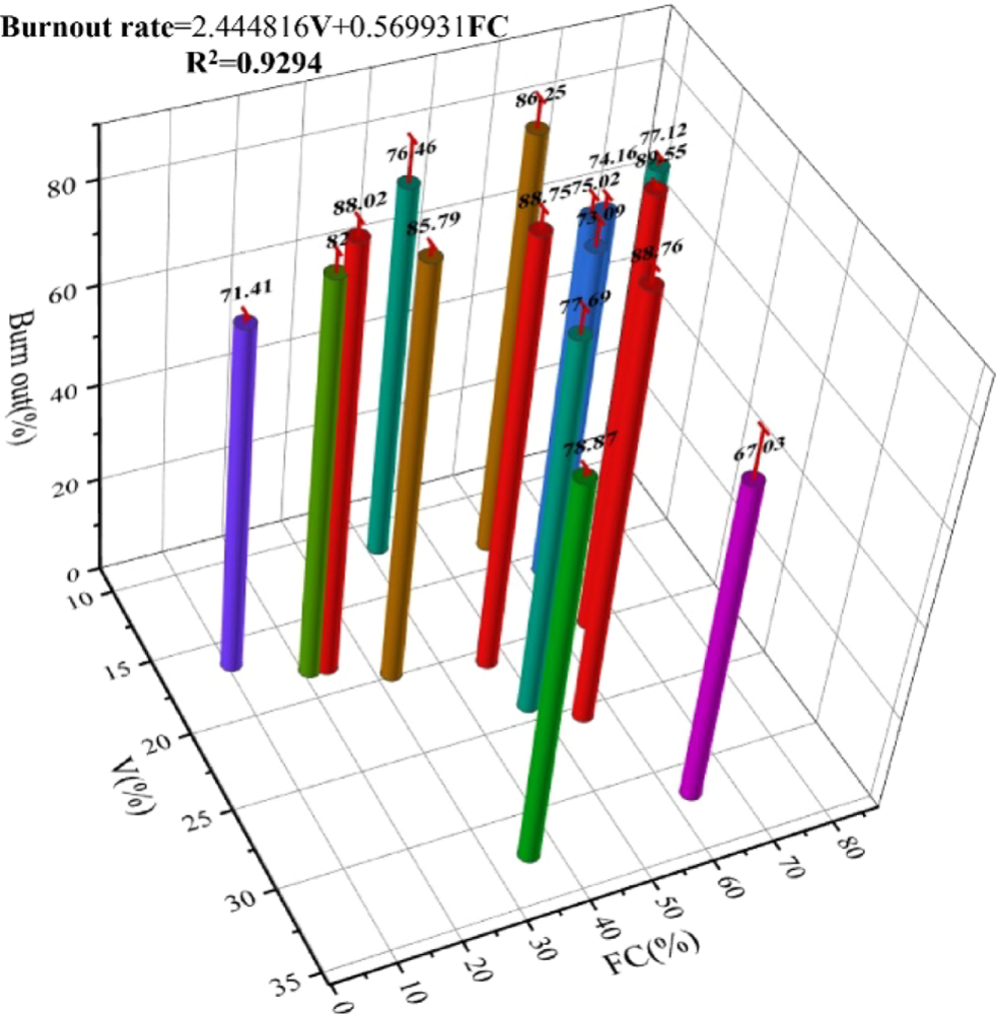
Burnout rate in relation to fixed carbon and volatiles.

The burnout rates of different biomass char samples are shown in Table 8. It can be seen that the burnout rates of different biomass char samples vary quite much; the burnout rate of most of the samples is above 70%; only the burnout rate of B8 is lower than 70%. B12 has the highest burnout rate, which is 89.55%. This may be because the ash melting characteristic temperature of B12 is lower than that of B8, so B12 burns more completely. The burnout rates of B6, B9, and B11–B15 were more than 80%, which were 86.25, 82.00, 85.79, 89.55, 88.75, 88.76, 88.02, 88.75%, respectively. Since there is no good linear relationship between volatiles (V), fixed carbon (FC) and burnout rate, fitting software was adopted to carry out linear fitting for the three, and the relationship diagram between volatiles, fixed carbon, and burnout rate is shown in Figure 14. It can be seen that the biomass char with a high burnout rate is mainly concentrated in the area where V is less than 25% and FC is less than 70%. Outside this area, there are only two kinds of biomass char samples, B1 and B8, whose burnout rates are 78.87 and 67.03%, respectively. With an increase of volatiles, the burnout rate increases gradually, and with an increase of fixed carbon, the burnout rate also increases gradually. The difference between the two values should not be too large, which may be because the higher volatiles correspond to lower fixed carbon, and there is not enough fixed carbon to support the combustion of biomass char when it enters the high-temperature reactor. For biomass char with relatively appropriate volatile and fixed carbon, their high volatile and has high fixed carbon in reaction with hot air, high volatile makes the reaction of biomass char fire in early time and can quickly burn, while high fixed carbon content can support continuous combustion reaction. In addition, the volatile and fixed carbon content should not be too high; a too high volatile content will lead to low fixed carbon content, resulting in less flammable substances, and a too high fixed carbon content will reduce the volatile content, resulting in the combustion performance of biomass char that is not good enough.

**Table 8:** Burnout rate of Different Biomass Char Samples (%)

sample	1	2	3	4	5	mean
B1	74.45	80.71	86.56	78.33	77.58	78.87
B2	70.40	75.58	68.03	73.41	70.43	71.41
B3	65.03	75.40	85.09	78.61	77.35	77.12
B4	67.89	84.27	72.06	79.26	81.74	77.69
B5	64.04	74.95	67.35	76.98	80.32	73.09
B6	79.93	89.63	89.18	95.10	79.72	86.25
B7	67.10	81.01	63.26	81.26	82.70	76.46
B8	55.92	72.19	54.99	72.99	88.16	67.03
B9	78.29	88.34	72.89	81.44	86.26	82.00
B10	71.50	74.93	67.06	78.63	89.36	75.02
B11	75.92	86.07	83.49	87.82	91.37	85.79
B12	90.48	89.21	84.27	88.95	93.18	89.55
B13	95.05	92.11	75.14	85.51	88.67	88.76
B14	91.61	97.40	82.19	86.92	85.52	88.02
B15	84.79	89.18	83.56	92.29	95.17	88.75
B16	75.87	89.27	71.15	75.46	69.31	74.16

Therefore, it can be inferred that the burnout rate is related to both the volatiles and fixed carbon, and the influence of volatiles on the burnout rate is greater than that of fixed carbon. In addition, due to the large density difference in biomass char samples, the contact time between the biomass char and hot air after it is flushed into the high-temperature reactor by high-pressure gas is different, which may also lead to the large difference in the burnout rate of different biomass char samples.

## 5 Conclusions

The fixed carbon, volatile content, and high heating value of the biomass char in this study are between bituminous coal and anthracite, and most of the biomass char samples have higher ash and moisture contents. However, the content of alkali metal and alkaline earth metal in biomass char is relatively high, so the biomass char needs to be dealkalized before use. All of the biomass char samples have good grindability and fine particle size. It can be seen that the ignition point of most biomass char samples is between those of bituminous coal and anthracite, and some biomass char samples are weakly explosive; with an increase of volatile matter, the ignition point and explosiveness show a downward and upward trend, respectively. Through the study of ash melting characteristics, it is found that the ash melting point of biomass char was generally low and the softening temperature (ST) decreased with an increase of CaO content and increased with an increase of SiO_2_ content. The combustibility experimental study finds that most of the biomass char samples had good combustion performance, and the reaction could be completed before 700 °C.

Finally, the combustion behavior of biomass char in blast furnace tuyere raceway was simulated and the burnout rate was measured. The results showed that that the burnout rate is related to both the volatiles and fixed carbon and the influence of volatiles on the burnout rate is greater than that of fixed carbon. The burnout rates of B3 and B8 were 77.12 and 67.03%, respectively. Above all, B3 and B8 had good properties, but the burnout rate of B3 was higher, so B3 had the feasibility of applying to blast furnace injection, which indicates that woodblock char has the potential to be used as blast furnace injection fuel.
